# Pulmonary Cryptococcosis Infection in Non‐HIV Patients in a Tertiary Care Center in Taiwan

**DOI:** 10.1155/pm/4389033

**Published:** 2026-02-25

**Authors:** Kuan-Chieh Tu, Ting-Chia Chang, Mei-I Sung, Chung-Han Ho, Kuo-Chen Cheng, Wen-Liang Yu

**Affiliations:** ^1^ Department of Internal Medicine, Chia-Li Chi Mei Hospital, Tainan, Taiwan; ^2^ Department of Internal Medicine, Chi Mei Medical Center, Tainan, Taiwan, chimei.org.tw; ^3^ Department of Respiratory Therapy, Chi Mei Medical Center, Tainan, Taiwan, chimei.org.tw; ^4^ Department of Medical Research, Chi Mei Medical Center, Tainan, Taiwan, chimei.org.tw; ^5^ Department of Information Management, Southern Taiwan University of Science and Technology, Tainan, Taiwan, stust.edu.tw; ^6^ Department of Intensive Care Medicine, Chi Mei Medical Center, Tainan, Taiwan, chimei.org.tw; ^7^ Department of Medicine, School of Medicine, College of Medicine, Taipei Medical University, Taipei, Taiwan, tmu.edu.tw

**Keywords:** antifungal therapy, HIV-negative, pulmonary cryptococcosis

## Abstract

**Background:**

Pulmonary cryptococcosis is an important opportunistic fungal infection in immunocompromised individuals, including those with an infection of the human immunodeficiency virus (HIV) but it can be increasingly seen in non‐HIV patients.

**Methods:**

We retrospectively reviewed the medical records of 58 non‐HIV‐infected patients with International Classification of Diseases, Ninth Revision, Clinical Modification Code B45.0 (pulmonary cryptococcosis) who were admitted to the Chi‐Mei Hospital, Taiwan from January 2016 to April 2022.

**Results:**

Of the enrolled 58 cases, 56 patients had no evidence of disease outside the lungs, and only two patients (3.4%) had disseminated diseases. Thirty‐nine patients had pathologically confirmed pulmonary cryptococcosis, and 19 patients had clinically confirmed disease. Pulmonary cryptococcus patients with cancer had a nonsignificant numerically higher rate of ICU use (14.29% vs. 5.13%; *p* = 0.348). There were similar mortality rates in both cancer and noncancer patients with pulmonary cryptococcosis (4.76% vs. 2.70%; *p* = 0.581). Patients in the cancer and noncancer groups had a similar duration of hospital stay (7 vs. 6 days, *p* = 0.799) and low mortality rates (4.76% vs. 2.70%, *p* = 0.581) on standard antifungal therapy.

**Conclusions:**

Cancer and noncancer patients had similar good outcomes after receiving appropriate standard antifungal treatment. Asymptomatic patients with pulmonary cryptococcosis diagnosed incidentally are self‐limited and require no antifungal therapy.

**Trial Registration:**

IRB number: 11111‐004.

## 1. Introduction

Cryptococcosis is an infectious disease caused by *Cryptococcus* species, especially *Cryptococcus neoformans* and *Cryptococcus gattii* [1]. Pathogenic cryptococci are encapsulated aerobic yeast and can be divided into four different serotypes according to capsular agglutination reactions [2]. The fungi exist in the environment such as pigeon droppings and eucalyptus trees and can infect humans via being inhaled [3]. When the fungi reach the alveoli, it would interact with macrophage in the lung initially and may lead to pulmonary cryptococcosis if the host is under poor immune status [4]. The fungi may even invade into the bloodstream, causing fungemia and cross the blood–brain barrier (BBB), leading to the infection of the central nervous system (CNS) and meningoencephalitis [5].

Pulmonary cryptococcosis is the most common manifestation among patients with cryptococcosis, and other infection sites include CNS, skin, eye, and prostate infection [6]. The diagnosis of the *Cryptococcus* infection typically depends on microscopy, serology, and culture studies. Cryptococcal antigen is an important biomarker for auxiliary diagnosis of *Cryptococcus* infection. However, sera from patients with pulmonary cryptococcosis are rarely positive in the absence of disseminated disease [7]. Another diagnostic method of pulmonary cryptococcosis is directly recognizing the pathogenic fungi through histologic staining of tissue samplings [8].

Worldwide, cryptococcal infection is typically associated with immunocompromised individuals such as patients with AIDS, immunosuppressive agent usage, organ transplantation, malignancies, and liver disease [9]. Cryptococcal infection is highly associated with HIV infection according to epidemiologic data [10]. Nevertheless, along with the development and progression of antiretroviral therapy, the incidence of cryptococcosis decreased gradually in developed countries [11, 12]. This epidemiological change makes other immunocompromised groups more important clinically; however, there are only limited data in other categories. Two large‐scale multicenter registries displayed that cryptococcosis accounted for 1.5% and 2.8% of invasive fungal infections, respectively, among patients with hematologic malignancy [13, 14]. Some authors even suggest the recent uses of more dose‐intense and lymphocyte‐depleting chemotherapeutic regimens further elevate the risk of cryptococcal disease in patients with malignancy [4, 15]. However, most investigations of cryptococcosis in patients with malignancy are concentrating on hematology malignancy [16]. Studies suggest that patients with a solid cancer also had a higher risk of disseminated cryptococcal infection and all‐cause mortality [17, 18]. In considering the increasing importance of cryptococcosis in patients with cancer, we assumed that cryptococcosis may cause a poor clinical outcome among patients with solid malignancy. Thus, we provided the clinical outcomes of active solid cancer patients with pulmonary cryptococcosis in a tertiary care center in Taiwan. The aim of this study is to evaluate the clinical outcome of pulmonary *Cryptococcus* infections in a tertiary care center in Taiwan. To our knowledge, this is the first study that compares the *Cryptococcus* infection among patients with and without active solid tumors in Taiwan.

## 2. Material and Methods

We conducted a single‐center, retrospective observational study of non‐HIV patients who were hospitalized with pulmonary cryptococcosis over the course of a 7‐year period (January 2016 to June 2022) at Chi‐Mei Medical Center in southern Taiwan. Chi‐Mei Medical Center is a nonprofit, 1288‐bed tertiary medical center in southern Taiwan. The study protocol was approved by the Ethics Committee of the Institutional Review Board of Chi‐Mei Medical Center in Taiwan. The need for written patient consent was waived because the study was retrospective in nature (IRB no. 11111‐004).

We identified patients with pulmonary cryptococcosis using ICD‐10‐CM Code B45.0. This study collected clinical data that consisted of type of cancer (gastrointestinal, breast, lung, hematologic, head and neck, and others); demographics; underlying diseases; need for stay in the intensive care unit (ICU); disseminated disease; and treatment regimens and clinical outcome (hospital stay, death, or survival). For the main analysis, patients were categorized into two groups: those with active cancer and those without active cancer (including patients with no history of malignancy and those with nonactive cancer). Information on underlying malignancy was obtained from oncology clinic notes, discharge summaries, pathology reports, and treatment records. Patients with documented solid or hematologic malignancy before or at the index hospitalization were classified according to prespecified criteria, whereas those without any history of malignancy were categorized as noncancer. Active cancer was defined a priori, consistent with prior studies, if at least one of the following criteria was met: (1) new diagnosis of malignancy within 6 months prior to the index hospitalization; (2) ongoing anticancer therapy (chemotherapy, targeted therapy, immunotherapy, radiotherapy, or cancer‐related surgery) within 6 months before admission or during hospitalization; (3) locally advanced, recurrent, or metastatic disease documented on imaging or clinical notes; (4) hematologic malignancy not in complete remission. Patients with a remote history of cancer not meeting these criteria (e.g., prior curative resection without recurrence and no recent therapy) were classified as nonactive cancer. By using the electronic medical records, we could identify 39 patients with pathologically proven pulmonary cryptococcosis, and 19 patients with clinically proven pulmonary cryptococcosis. Over the same span of time, we could only find two cases of AIDS patients with cryptococcal infections.

The majority of pulmonary cryptococcosis requires histological proof for a proven diagnosis. When surgical biopsy or resection is not available, others can be diagnosed clinically by radiographic findings and serum cryptococcal capsular polysaccharide antigen test. We excluded cases of HIV infection from the study population. Patients who were diagnosed with pulmonary cryptococcosis without pathological confirmation or cryptococcal antigen detection were not included.

Results were reported as percentages for categorical variables and median and interquartile range for continuous variables. We calculated the time from the diagnosis of pulmonary cryptococcosis to the all‐cause death or the last follow‐up date. Censored participants were those alive at the end of the follow‐up period on 31 August 2022.

All statistical analyses were conducted with SPSS 26. All assessed clinical information management was done using the Microsoft Office Excel program (2010 version).

## 3. Results

An overall 60 cases were screened in the current study and two were excluded since cryptococcal diagnoses were neither by histology nor by clinical definitions, leaving 58 cases for analysis. Of the 58 enrolled cases, 56 patients had no evidence of extrapulmonary diseases, and only two patients (3.4%) had disseminated disease. Thirty‐nine patients had pathologically confirmed pulmonary cryptococcosis, and 19 patients had clinically confirmed disease with radiographic findings and serum cryptococcal antigen test. We excluded two patients who were diagnosed neither by histology nor by clinical definitions.

The study population consisted of 32 men and 26 women, with a median age of 59.5 years (range, 49.75–67 years). Only two of the patients showed CNS involvement. Table 1 shows the demographic and clinical characteristics of all enrolled active cancer and noncancer patients from 2016 to 2022.

**Table 1:** Characteristics of patients with pulmonary cryptococcosis in active cancer and noncancer patients (*n* = 58).

Variables	Active cancer patients (*n* = 16)	Noncancer patients (*n* = 42)	*p*
Age (years)	62.5	59.5	0.256
Gender, *n* (%)			0.919
Female, *n* (%)	7 (43.75)	19 (45.24)	
Male, *n* (%)	9 (56.25)	23 (54.76)	
Primary cancer site			
Gastrointestinal, *n* (%)	7 (33.33)		
Breast, *n* (%)	3 (14.29)		
Lung, *n* (%)	4 (19.05)		
Hematologic, *n* (%)	1 (4.76)		
Head and neck, *n* (%)	4 (19.05)		
Other, *n* (%)	2 (9.52)		
Underlying disease, *n* (%)			
Congestive heart failure	0 (0)	2 (4.76)	
Coronary artery disease	2 (12.50)	1 (2.38)	0.181
DM	7 (43.75)	15 (35.71)	0.573
RA	0 (0)	2 (4.76)	
SLE	0 (0)	0 (0)	
COPD	0 (0)	3 (7.14)	
Liver disease	0 (0)	2 (4.76)	
ESRD	0 (0)	3 (7.14)	
Organ transplant	0 (0)	2 (4.76)	
Diagnostics, *n* (%)			0.437
Biopsy and pathology	12 (75.00)	27 (64.29)	
Serum CrAg Test	4 (25.00)	15 (35.71)	
ICU admission	2 (12.50)	3 (7.14)	0.424
CNS involvement	1 (6.25)	1 (2.38)	0.479

*Note:* Expressed as median and interquartile range or proportion of patients (%).

Abbreviations: CNS, central venous system; COPD, chronic obstructive pulmonary disease; CrAg, cryptococcal antigen; DM, diabetes mellitus; ESRD, end‐stage renal disease; RA, rheumatoid arthritis; SLE, systemic lupus erythematosus.

Comorbidities were equally distributed between the two groups. The most common solid cancer was gastrointestinal cancer, followed by lung, head and neck, and breast. Significant differences in disseminated disease and ICU admission were not observed.

Treatment regimens and clinical outcome data are shown in Table 2. Most patients received fluconazole monotherapy (*n* = 38, 65.52%), followed by amphotericin B plus fluconazole (*n* = 3, 5.17%) as induction therapy for disseminated cryptococcosis. The median treatment duration was 188 days and 124 days for active cancer and noncancer patients, respectively. Sixteen patients received no antifungal therapy for an asymptomatic pulmonary cryptococcal infection. Of the untreated patients, 12 were pathologically confirmed and four were diagnosed by radiographic findings in combination with serum antigen testing, whereas 15 clinically defined cases were symptomatic with fever, cough, or dyspnea. Patients in the active cancer and nonactive cancer groups had similar durations of hospital stay (7 vs. 5.5 days) and low mortality rates (6.25% vs. 2.38%, *p* = 0.581).

**Table 2:** Treatment and clinical outcomes for patients with pulmonary cryptococcosis.

Variables	Active cancer patients (*n* = 16)	Noncancer patients (*n* = 42)	*p*
Treatment			0.468
Fluconazole mono	9 (56.25)	29 (69.05)	
Amphotericin B + fluconazole	1 (6.25)	2 (4.76)	
Amphotericin B mono	0 (0)	0 (0)	
Itraconazole + fluconazole	1 (6.25)	0 (0)	
No	5 (31.25)	11 (26.19)	
Duration of antifungal treatment	188	124	
Hospital stay	7	5.5	
Mortality	1 (6.25)	1 (2.38)	0.724

*Note:* Expressed as median or proportion of patients (%).

## 4. Discussion

In this retrospective case‐control study using single‐center clinical data, 60 cases were initially identified, of which two were excluded, leaving 58 patients with *Cryptococcus* infection from 2016 to 2022. Under standard antifungal therapy, we found that patients with active cancer had a mortality rate comparable to those without cancer.

Pulmonary cryptococcosis is an opportunistic infection typically found in patients with immunocompromised status, especially those with T cell mediated deficiencies in AIDS patients [19]. In the malignancy population, most discussions and publications regarding *Cryptococcus* infection focused on patients with hematologic malignancy [4, 16, 20]. Our study mainly involved different populations. Cryptococcosis is not considered a typical opportunistic infection in the overall cancer population, in whom neutropenia is the predominant form of immune compromise [4]. However, a study displayed that malignant solid tumors are also associated with a higher risk of recurrent pulmonary cryptococcosis and mortality from any cause when compared with those without any comorbidity [18]. In our study, the results demonstrated that pulmonary cryptococcosis among patients with active malignancy with appropriate therapy had similar clinical outcomes as those without active cancer, which resembled the observation of prior studies that focused on the non‐HIV populations [21, 22].

In our study, solid malignancy includes head and neck cancers (*n* = 4), breast cancers (*n* = 3), lung cancers (*n* = 4), GI tract cancers (*n* = 7), GU tract, and GYN cancers (*n* = 2). Males were prone to be infected in both groups, and this observation is similar to previous studies [21, 22]. Chin Lin et al. displayed that diabetes mellitus and autoimmune disease are associated with higher recurrence rates and all‐cause mortality [18]. In our study, there was no statistical difference in the comorbidity between the active cancer group and the nonactive cancer group. Though the flourishing development of the auxiliary diagnosis of cryptococcosis (CrAg and FlimArray), there is still a lack of a gold standard noninvasive diagnostic method for pulmonary cryptococcosis. Biopsy and histological tissue‐proven remain the gold standard diagnosis of pulmonary cryptococcosis [21, 23]. In the presenting study, over two‐thirds of populations diagnosed with pulmonary cryptococcosis were based on biopsy tissue proven, and others were basically on the radiography findings and serum cryptococcal antigen. The ratio of tissue proof of pulmonary cryptococcosis was higher than prior studies [18, 21, 24].

In our study, there is no statistically significant difference in cryptococcal meningitis between the two groups (6.25% vs. 2.38%, *p* = 0.479), and the same trend was noted on the issue of ICU admission rate (12.5% vs. 7.14%, *p* = 0.424). There is no statistical difference in the ratio of patients accepting the standard treatment therapy between both groups, and the percentage is 68.75% and 73.80%, respectively. Once pathogenic *Cryptococcus* invades the bloodstream and gives rise to disseminated infection, it could cause extrapulmonary site infection and even break through the BBB and contribute to cryptococcal meningitis. It is believed that cryptococcal meningitis is highly associated with patients with cancer and is a poor prognosis factor and would increase the mortality [4]. Nevertheless, in our study, there were a little bit more cryptococcal meningitis and ICU admission in the active cancer group without statistical difference; both groups had good prognoses under appropriate standard therapy.

The treatment strategies for cryptococcal infection in our study are based on the evidence‐based guideline available from the Infectious Diseases Society of America (IDSA) and the Australasian Antifungal Guidelines Steering Committee [25, 26]. Induction therapy with amphotericin B and flucytosine/fluconazole, consolidation and maintenance therapy with fluconazole comprised the backbone of the treatment strategy of cryptococcal meningitis and severe pulmonary cryptococcosis among immunocompromised patients. Fluconazole for 6–12 months was suggested for mild to moderate pulmonary cryptococcosis and prophylactic antifungal therapy for patients with asymptomatic antigenemia is still debated [25]. In our study, 68.7% of the active cancer group and 73.80% of the nonactive cancer group had accepted standard therapy. Monotherapy with fluconazole accounted for 56.25% and 69.04% of the population in the active cancer group and nonactive cancer group, respectively. Of our patients, only 3.45% experience extrapulmonary infection, and this is much lower than that seen in patients with HIV [27]. Within patients with more severe pulmonary cryptococcosis and cryptococcal meningitis, amphotericin B plus flucytosine is regarded as standard therapy. The proportion of the usage of amphotericin B plus flucytosine is 6.25% and 4.76% in the active cancer group and nonactive cancer group, respectively. The treatment duration is longer in the cancer group (188 days vs. 124 days) and the overall mortality rate between the two groups is approximately 6.25% and 2.38%, respectively. The mortality rate of pulmonary cryptococcosis varies widely and is dependent on the host factors including comorbidity, immunity, and the extent of infection [28]. From our data, the mortality rate during admission is low and this can be partially explained by the disease severity in our study is relatively less. Previous studies reported a much higher mortality rate in non‐HIV patients with pulmonary cryptococcosis, but it is notable that these reported high mortality rates are also associated with disseminated infections [29, 30]. Our data suggested that pulmonary cryptococcosis among patients with active cancer could be successfully controlled through standard antifungal therapy, and this observation is similar to a prior report [18]. In this cohort, 16 patients were asymptomatic and did not receive antifungal treatment. The decision to treat pulmonary cryptococcosis was primarily based on the attending physician′s clinical judgment, taking into account the patient′s comorbidities, immune status, and disease progression.

There are certain limitations to the present study, with the main limitation being its retrospective nature at specific time points. Second, this study is based on a single‐center database, which carries inherent limitations regarding the generalizability of our findings, including differences in patient characteristics, referral patterns, management strategies, and the relatively small sample size. Third, our study was based on the clinical data in a tertiary hospital, and we used the ICD code for patient collection, which might have some limitations as follows: The infection site of *Cryptococcus* with the ICD‐9 diagnostic code is too ambiguous and unspecific, and thus we used ICD‐10 for patient collection from 2016 to 2022. The dataset is based on the accuracy of claim‐based diagnosis using ICD‐10 administration. We improved the accuracy of diagnosis coding by providing diagnostic methods for patients with pulmonary cryptococcosis. Approximately one‐third of patients were diagnosed as pulmonary cryptococcosis via serum cryptococcal antigen data, and we could not avoid false positive data. Forth, a key limitation of our study is that the clinical context behind hospitalization and treatment decisions cannot be fully determined from this retrospective dataset. Sixteen asymptomatic patients (27.6%) were hospitalized for abnormal chest radiographs but did not receive antifungal therapy, and similarly, six cancer patients with biopsy‐proven pulmonary cryptococcosis were untreated. The reasons for these decisions cannot be clarified in the current study. These patterns likely reflect heterogeneity in clinical judgment, undocumented contraindications, or patient factors not captured in the database. Therefore, caution is needed when interpreting the proportion of untreated cases, as our data may underestimate the true treatment rate for clinically significant cryptococcosis.

## 5. Conclusion

In conclusion, active cancer and nonactive cancer patients had similar good outcomes after receiving appropriate standard antifungal treatment. Asymptomatic pulmonary cryptococcal infections diagnosed incidentally are self‐limited and usually do not require antifungal therapy.

**Nomenclature**
AIDS: Acquired Immune Deficiency Syndrome
CNS: central nervous system
ICD‐10: International Statistical Classification of Diseases and Related Health Problems 10th Revision
ICU: intensive care unit
HIV: human immunodeficiency virus

## Author Contributions

K‐C. Cheng and W‐L.Y., the guarantors of this manuscript. W‐L.Y., K‐C. Cheng, K‐C. Tu, and T‐C.C. contributed to the conception and design of the study. C‐H.H. assisted the software operation. M‐I.S. analyzed and interpreted the data. K.C. Tu and T‐C.C. drafted the manuscript. W‐L.Y. and K‐C. Tu edited the final manuscript. K‐C. Tu and T‐C.C. contributed equally to this work and are designated as co–first authors.

## Funding

No funding was received for this manuscript.

## Disclosure

All authors have read and agreed to the published version of the manuscript.

## Ethics Statement

The study was conducted according to the guidelines of the Declaration of Helsinki, and approved by the Research Ethics Committee of Chi Mei Hospital (IRB no. 11111‐004).

## Consent

No individual′s data are included in this study.

## Conflicts of Interest

The authors declare no conflicts of interest.

## Data Availability

The data and materials of this study are available from the corresponding author upon reasonable request.
